# Risk factors for postoperative complications following one-stage proximal hypospadias repair involving the disconnection of the urethral plate: a retrospective study

**DOI:** 10.1186/s12887-023-04339-w

**Published:** 2023-10-07

**Authors:** Jianjun Hu, Yaowang Zhao, Tianqu He, Yifu Chen, Zhaohui Wang, Liucheng Peng

**Affiliations:** Department of Urology, Hunan Children’s Hospital, Changsha, 410007 Hunan China

**Keywords:** Proximal hypospadias, Disconnection of the urethral plate, Complications, Length of the reconstructed urethra, Glans width, Risk factors

## Abstract

**Background:**

Children with hypospadias are at risk of serious physical and mental health problems, including abnormal urination, sexual dysfunction, and infertility. The sole available treatment is the surgical restoration of genital appearance and function. Proximal hypospadias (PH) correction is more challenging and carries a higher risk of complications than does distal hypospadias correction, with a higher likelihood of postoperative complications requiring additional surgery, resulting in considerable economic and psychological strain for families. Herein, we aimed to identify factors associated with complications following one-stage PH repair with urethral plate disconnection.

**Methods:**

We retrospectively analyzed data from 236 children who underwent PH repair at our center between December 2020 and December 2022. We collected information on age, surgical procedure, length of the reconstructed urethra (LRU), glans width (GW), ventral curvature, surgical approach, preoperative androgen use, suture type, presence of prostatic utricle, body mass index, season of surgery, anesthesia type, low birth weight, preterm birth, follow-up period, and complications. Surgical complications included urethral fistulas, urethral diverticula, anastomotic strictures, urethral strictures, glans dehiscence, and penile curvature recurrences. The study population was divided into complication and no-complication groups, and univariate and multivariate analyses were conducted.

**Results:**

Of the 236 patients with PH who had a median follow-up of 10.0 (8.0, 14.0) months, 79 were included (33.5%) in the complication group and 157 were included (66.5%) in the no-complication group. In the univariate analysis, age (*P* < 0.001), LRU (*P* < 0.001), degree of penile curvature (*P* = 0.049), and PH with prostatic utricle (*P* = 0.014) were significantly associated with complications after PH repair. In the multivariate logistic regression analysis, LRU (*P*<0.001, odds ratio [OR] = 3.396, 95% confidence interval [CI]: 2.229–5.174) and GW (*P* = 0.004, OR = 0.755, 95%CI: 0.625–0.912) were independent factors influencing postoperative complications. The optimal LRU threshold was 4.45 cm (area under the curve, 0.833; sensitivity, 0.873; specificity, 0.873; *P*<0.001, OR = 3.396, 95% CI: 2.229–5.174).

**Conclusions:**

LRU and GW are independent factors affecting PH complications. An LRU of < 4.45 cm and an increased GW can reduce the risk of complications.

## Background

Hypospadias is a common malformation among male children, with an incidence of approximately 9.03 per 10,000 in China [1]. It can be classified as distal, and proximal hypospadias (PH) [2]. In PH, the urethral orifice is located at the penile-scrotal junction, scrotum, or perineum [3], and PH accounts for approximately 20% of all hypospadias [4]. Children with hypospadias and their families are at risk of serious physical and mental health problems. The only available treatment for hypospadias is surgical restoration of genital appearance and function. The treatment objective is to restore normal urination, help patients achieve a healthy sexual life and fertility, and eliminate patients’ psychological burden [5]. Compared to distal hypospadias correction, PH correction is more challenging and is associated with a higher risk of complications, including postoperative complications such as urethral fistulas, anastomotic strictures, urethral strictures, urethral diverticula, glans dehiscence, and recurrence of penile curvature [6]. Such postoperative complications may necessitate additional surgery, resulting in considerable economic and psychological strain for the patients’ families and society at large [7–9].

The existing surgical approaches for PH are controversial, with the most important debates being over preserving versus disconnecting the urethral plate and one- versus two-stage surgery. For PH treatment, Arshadi et al. [10] used the tubularized incised plate technique; in their study, all participants had mild penile curvatures. However, if the penile curvature exceeds 30° after foreskin removal, urethral plate retention is not appropriate. Most PH cases in China are associated with higher-grade penile curvature [11]. In the study by Snodgrass et al. [12], the complication rate for PH repair was 37%, and the recurrence rate of penile curvature was 26%. Braga et al. [13] compared two groups who underwent dorsal plication or urethral plate disconnection for PH correction. Penile curvature recurrence rates after surgery were 27.9% and 7.4%, respectively, with a high recurrence rate after urethral plate preservation. As PH is typically associated with penile curvature, urethral plate disconnection is necessary in PH treatment [14]. In terms of surgical stages, two-stage surgery requires higher surgical costs, anesthesia use, and number of operations, and the second surgery may increase the psychological burden on the child. Upon gaining experience and surgical skills, surgeons are able to perform one-stage hypospadias repair more effectively [15, 16], and our center recommends a one-stage repair for PH.

Wang et al. [17] revealed that in one-stage PH repair, Duplay procedure combined with transverse preputial island flap and the modified Koyanagi procedure were associated with complication rates of 40.0% and 50.0%, respectively. This is significantly higher than the complication rate of 11.1% associated with the two-stage approach. Reduction in complication rates of one-stage PH repair is challenging for pediatric urologists. However, research in this area is still lacking.

Surgeons at our center are experienced in one-stage PH repair with urethral plate disconnection. Both parents and physicians have been satisfied with the postoperative results, which involve fewer operations, less anesthesia use, and lower costs for patients.

Thus, this study aimed to identify factors associated with complications following one-stage PH repair with urethral plate disconnection. We retrospectively analyzed children with PH who underwent one-stage repair involving urethral plate disconnection. According to the presence or absence of postoperative complications, the children were divided into complication and no-complication groups. To investigate the risk factors associated with complications following PH surgery, univariate and multivariate analyses were conducted.

## Methods

### Patients

Herein, we retrospectively analyzed the data of 236 children with PH who underwent their first surgery between December 2020 and December 2022. The study was carried out in accordance with the tenets of the Declaration of Helsinki. The Ethics Committee of the Hunan Children’s Hospital approved the study (approval number: HCHLL-2023- 36), and the requirement for informed consent was waived,it was waived owing to the retrospective nature of the study.

### Definitions

The ventral curvature was preoperatively measured using an orthopedic protractor. A curvature of 30–45° was considered moderate and that of > 45° was considered severe [12]. Cystoscopy was used to identify the prostatic utricle.

Surgical complications included urethral fistulas, urethral diverticula, anastomotic strictures, urethral strictures, glans dehiscence, and penile curvature recurrences [2]. The no-complication group comprised patients who did not experience complications for more than 3 months postoperatively, whereas the complication group comprised those who experienced complications that required postoperative surgical intervention.

Inclusion criteria were: patients who underwent 1) their first surgery for PH and 2) one-stage treatment involving urethral plate disconnection using the Duckett, Duckett + Duplay, or modified Koyanagi procedures. The exclusion criteria were: 1) patients who had undergone failed urethroplasty procedures at other hospitals and required reoperation or those who had undergone one-stage urethroplasty, 2) those undergoing staged repair, 3) those with a mild penile curvature treated surgically to preserve the urethral plate, and 4) those with incomplete datasets.

### Surgical methods

Our center offers three types of one-stage urethroplasty through urethral plate disconnection: the Duckett [18], Duckett + Duplay [19], and modified Koyanagi [20] procedures. All surgical procedures were conducted by one experienced surgeon, who has performed over 100 operations annually for over 5 consecutive years. The surgical procedures are described below.

**Fig. 1:**
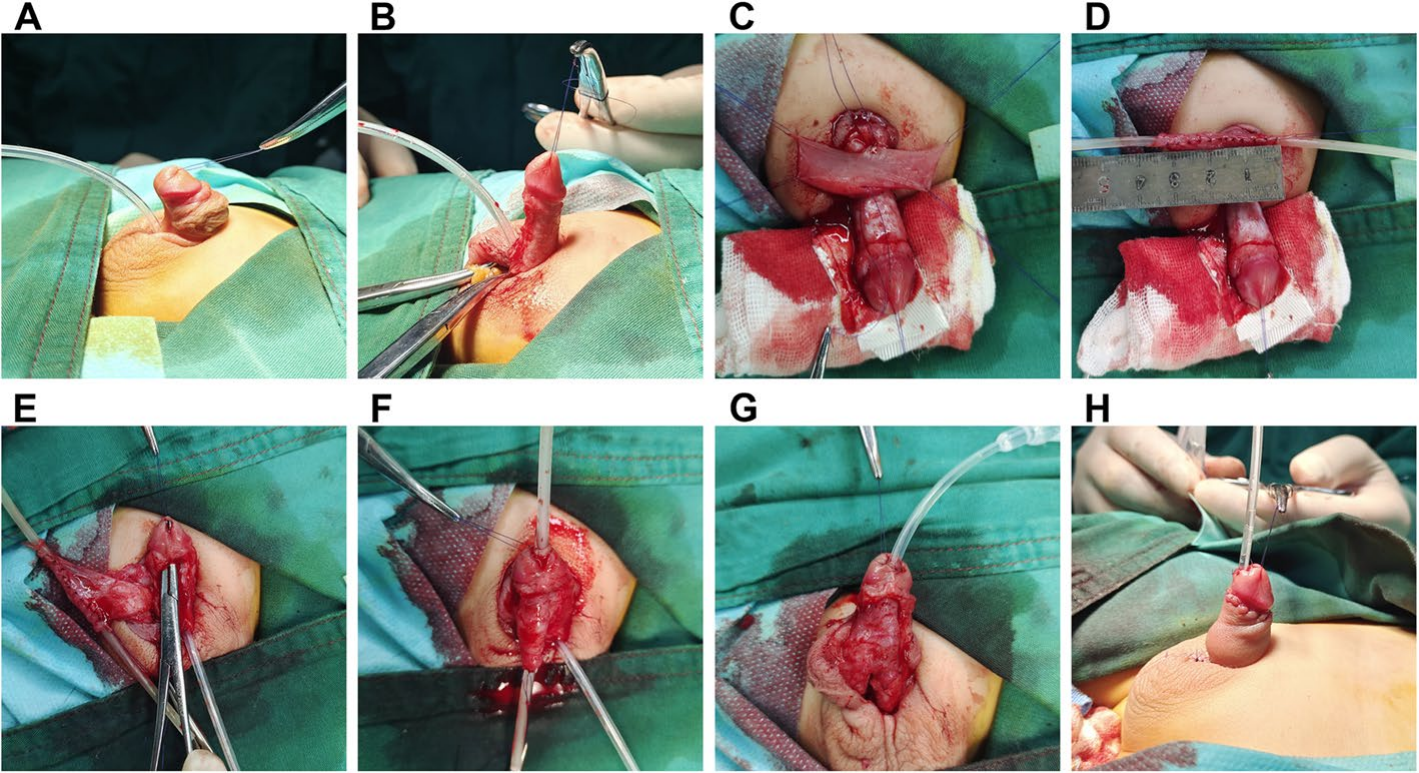
The Duckett procedure **A** Preoperative appearance **B** Correction of penile curvature through disconnection of the urethral plate **C** Taking the transverse pedicle rectangular preputial flap **D** Turning the longitudinal flap along the interrupted suture to form the new urethra **E** Dissection of the coronal groove of the penis head from the corpus cavernosum **F** Passing the new urethra through the glans; the new urethral orifice is formed at the end of the glans **G** The original urethral orifice is end-to-end anastomosed with the other end, and the new urethra is covered with tissue **H** Postoperative appearance

The Duckett procedure generally comprises urethroplasty of the transverse preputial flap and cutting of the fibrous chordae and the urethral plate. A penile erection test is performed for curvature correction, and penis dorsal plication is performed for uncorrected curvatures. The length of the missing urethra is measured, followed by excision of a rectangular flap from the inner foreskin plate. This flap is turned along the interrupted suture to form the new urethra. The coronal groove of the penis head is dissected from the corpus cavernosum, and the new urethra is passed through the glans. The new urethral orifice is formed at the end of the glans, the original urethral orifice is end-to-end anastomosed, and the new urethra is covered with tissue (Fig. 1).

**Fig. 2:**
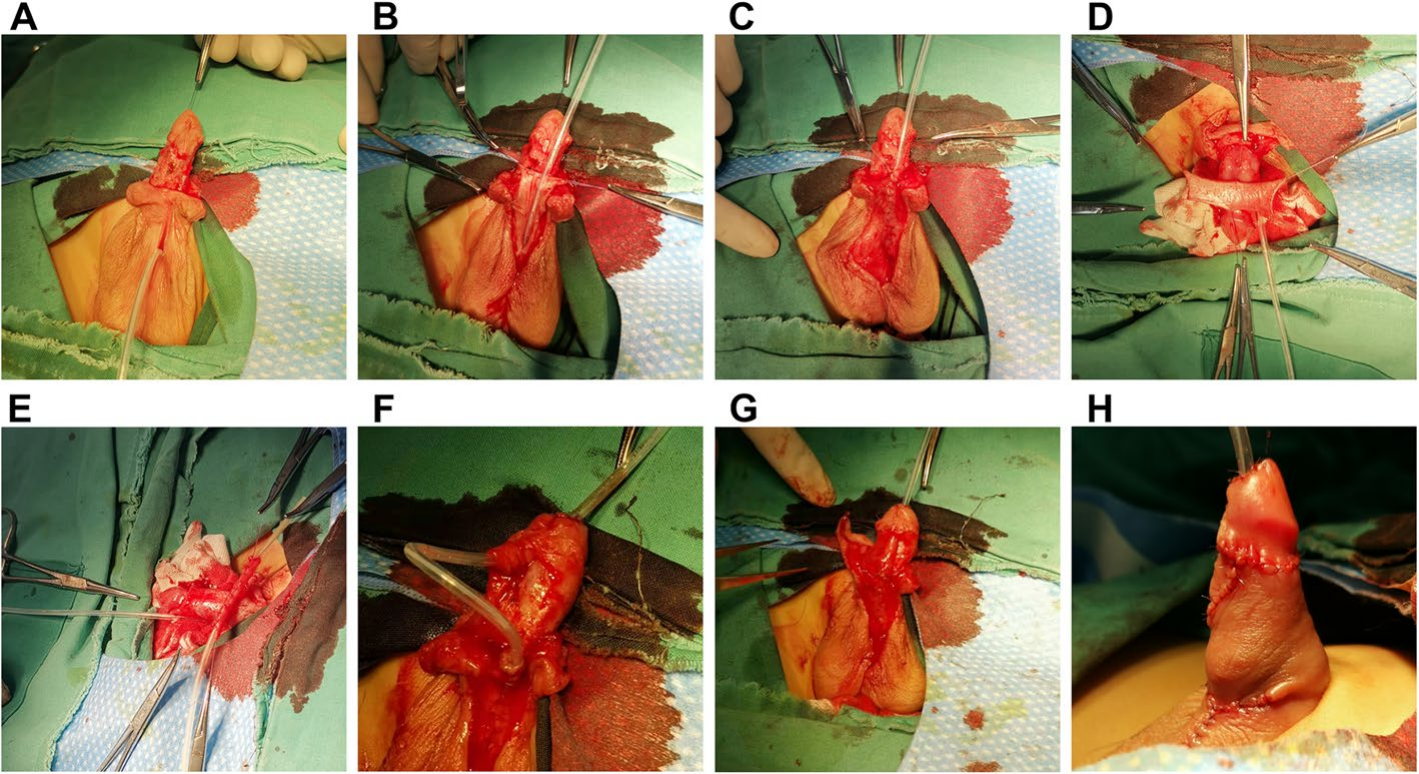
The Duckett + Duplay procedure **A** Correction of penile curvature through disconnection of the urethral plate **B** A U-shaped incision is made longitudinally in the scrotal skin around the urethral orifice **C** Taking the U-shaped rolled flap **D** Taking the transverse pedicle rectangular foreskin flap **E** Turning the longitudinal flap along the interrupted suture to form the new urethra **F** Passing the new urethra through the glans; the new urethral orifice is formed at the end of the glans **G** The original urethral orifice is end-to-end anastomosed with the other end and the new urethra is covered with tissue **H** Postoperative appearance

The Duckett + Duplay procedure is based on the Duckett procedure. A U-shaped incision is made longitudinally in the scrotal skin around the urethral orifice, and the urethra is formed by separating the tubed flap. The urethra is then linked to the inclined plane of the interrupted anastomosis of the urethra of the pedicle preputial flap to form the new urethra that is covered with tissue (Fig. 2).

**Fig. 3:**
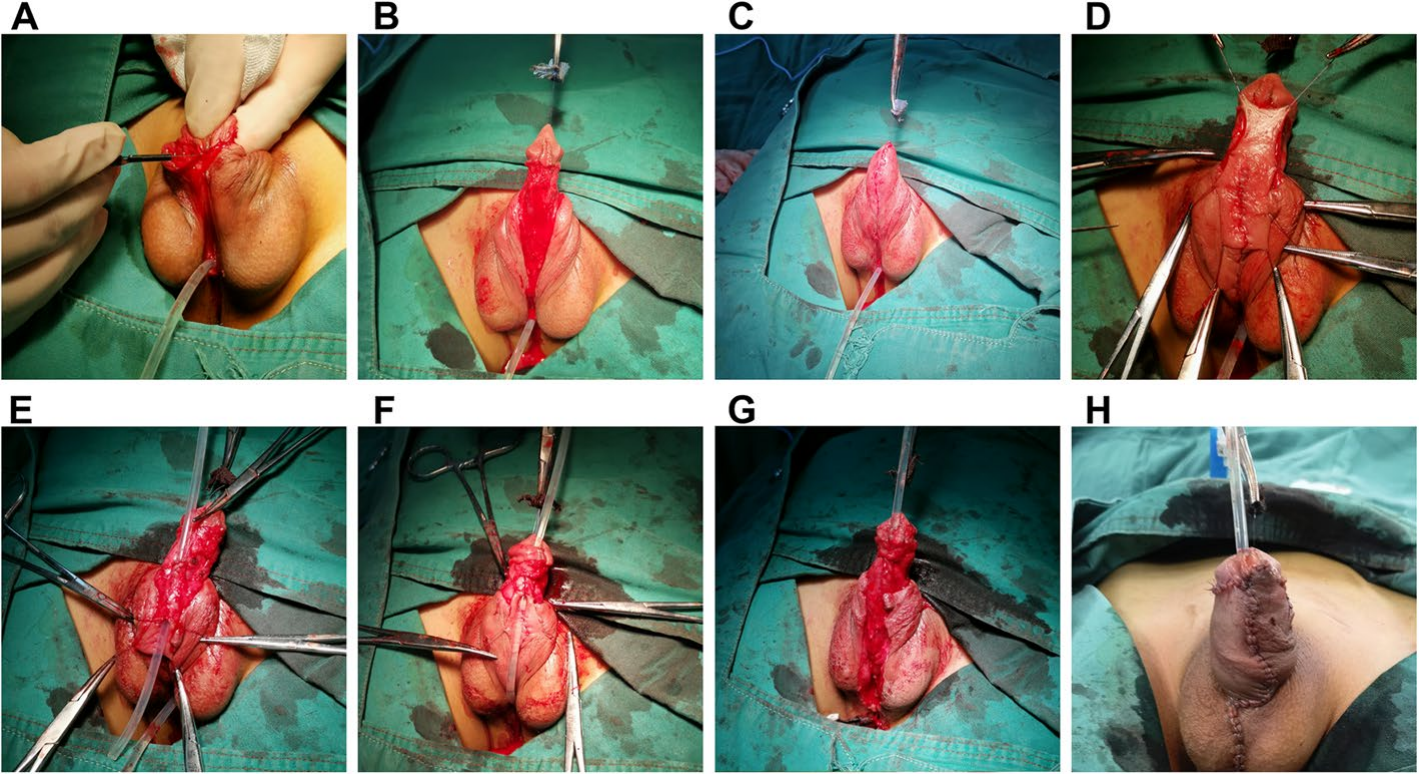
The modified Koyanagi procedure **A** Correction of penile curvature through disconnection of the urethral plate **B** Correction of penile curvature. The Y-shaped flap is visible **C** Suturing the Y-shaped flap **D** Cutting the rectangular flap **E** Longitudinal suture of the dermal surface of the rectangular flap to form the new urethra **F** Passing the new urethra through the glans; the new urethral orifice is formed at the end of the glans **G** The distal urethra is formed, and the new urethra is covered with tissue **H** Postoperative appearance

In the Modified Koyanagi procedure, the fibrous chordae and urethral plate are first cut. A penile erection test is performed for curvature correction, and penis dorsal plication is performed for uncorrected curvatures. The length of the missing urethra is measured, after which the penile scrotum is linked with a Y-shaped flap from the pedicle. This flap is sutured into a rectangular flap, and the dermal surface of the rectangular flap is sutured longitudinally to form the new urethra. The coronal groove of the penis head is dissected from the corpus cavernosum, the new urethra is passed through the glans. The new urethral orifice is formed at the end of the glans, the original urethral orifice is end-to-end anastomosed, and the new urethra is covered with tissue (Fig. 3).

In this study, the penis was tightly wrapped with silver ion and Vaseline gauze dressings postoperatively, and silicone catheters were routinely used to drain urine. The outer dressing was removed one week following surgery, and the patient was discharged. The catheter was removed in-hospital 4 weeks after surgery.

### Data collection

The hospital electronic medical record system was used to obtain data on the study participants’ age, body mass index, birth weight (to check if this was low), incidence of preterm birth, preoperative use of androgens, type of anesthesia, surgical approach and procedure, length of the reconstructed urethra (LRU), glans width (GW), degree of penile curvature, type of suture, presence of prostatic utricle, season of surgery, and complications. Complications comprised postoperative urethral fistulas, opening urethral strictures, anastomotic strictures, urethral diverticula, penile re-bending, and glans dehiscence.

### Statistical analysis

IBM SPSS Statistics 25.0 software (IBM Corp., Armonk, NY, USA) was used for all statistical analyses. After assessing normality with the Shapiro–Wilk test, mean ± standard deviation values were used to describe variables conforming to normality, median and interquartile ranges (P25, P75) to describe other continuous variables, and the composition ratio to describe categorical variables. The t-test was used to compare the means of quantitative data conforming to normality between two groups, the rank sum test to compare the means of other quantitative data between two groups, and the chi-square test to compare rates between two groups. Variables with *P* < 0.2 in the univariate analysis were included in the multivariate logistic regression analysis. Receiver operating characteristic curves were used to assess independent influences on predicting complication risk following hypospadias surgery. A test level of α = 0.05 was used, and *P* < 0.05 was considered statistically significant.

## Results

### Study population

Between December 2020 and December 2022, 236 children with PH were treated at our hospital, with an average age of 3.0 (2.0, 4.4) years and a median follow-up period of 10.0 (8.0, 14.0) months. In total, 124 PH cases were treated using the Duckett procedure, 62 using the Duckett + Duplay procedure, and 50 using the modified Koyanagi procedure. Postoperative complications occurred in 79 cases (33.5%), including 35 cases (44.3%) of urethral fistulas, 5 cases (6.3%) of anastomotic strictures, 20 cases (25.3%) of urethral strictures with diverticula, 10 cases (12.7%) of urethral diverticula, 6 cases (7.6%) of urethral strictures with urethral fistulas, and 3 cases (3.8%) of anastomotic strictures with urethral diverticula. Recurrences of glans dehiscence or penile curvature were not recorded.

### Univariate analysis

Significant differences were observed in the incidence of postoperative complications in the univariate analyses for age (*P*<0.001), LRU (*P*<0.001), degree of penile curvature (*P* = 0.049), and PH with prostatic utricle (*P* = 0.014) (Table 1).

**Table 1:** Univariate analysis results

Variable	Total	Group with postoperative complications (*n* = 157)	Group without postoperative complications (*n* = 79)	Z/χ^2^	*P*-value
Age (years), M (P25, P75)	3.0 (2.0, 4.4)	2.3 (1.8, 3.5)	4.2 (3.2, 8.2)	-6.33	<0.001
Length of the reconstructed urethra (cm), M (P25, P75)	4.5 (3.5, 5.1)	4.0 (3.2, 4.7)	5.2 (4.6, 6.2)	-8.36	<0.001
Glans width (mm), M (P25, P75)	14.0 (13.0, 16.0)	14.0 (13.0, 16.0)	14.0 (12.0, 15.0)	-1.89	0.059
BMI (kg/m^2^), M (P25, P75)	15.9 (14.9, 17.1)	15.9 (14.9, 16.8)	15.9 (14.9, 17.6)	-0.69	0.489
Follow-up time (months), M (P25, P75)	10.0 (8.0, 14.0)	10.0 (8.0, 14.0)	10.0 (8.0, 12.0)	-1.11	0.269
Degree of penile curvature, n (%)					
Moderate (30–45°)	67	51 (76.1)	16 (23.9)	3.87	0.049
Severe (> 45°)	169	106 (62.7)	63 (37.3)		
Surgical method, n (%)					
Duckett	124	86 (69.4)	38 (30.6)	1.02	0.601
Duckett + Duplay	62	40 (64.5)	22 (35.5)		
Modified Koyanagi	50	31 (62.0)	19 (38.0)		
Preoperative androgen use, n (%)					
No	169	111 (65.7)	58 (34.3)	0.19	0.662
Yes	67	46 (68.7)	21 (31.3)		
Suture type, n (%)					
Monofilament	143	98 (68.5)	45 (31.5)	0.66	0.418
Multifilament	93	59 (63.4)	34 (36.6)		
PH with prostatic utricle, n (%)					
No	162	116 (71.6)	46 (28.4)	5.99	0.014
Yes	74	41 (55.4)	33 (44.6)		
Season of surgery, n (%)					
Spring	65	48 (73.8)	17 (26.2)	6.59	0.086
Summer	55	29 (52.7)	26 (47.3)		
Autumn	83	57 (68.7)	26 (31.3)		
Winter	33	23 (69.7)	10 (30.3)		
Anesthesia mode, n (%)					
General anesthesia + sacral anesthesia	173	116 (67.1)	57 (32.9)	3.08	0.215
General anesthesia + epidural anesthesia	48	34 (70.8)	14 (29.2)		
General anesthesia + penile nerve block	15	7 (46.7)	8 (53.3)		
Low birth weight, n (%)					
No	163	111 (68.1)	52 (31.9)	0.59	0.444
Yes	73	46 (63.0)	27 (37.0)		
Preterm infants, n (%)					
No	161	108 (67.1)	53 (32.9)	0.07	0.791
Yes	75	49 (65.3)	26 (34.7)		

*Abbreviations*: *BMI* body mass index, *M*,median, (P25, P75), interquartile range

### Multivariate analysis

Multivariate logistic regression analysis involving variables with *P* < 0.2 in univariate analysis revealed that LRU (*P*<0.001, odds ratio [OR] = 3.396, 95% confidence interval [95%CI]: 2.229–5.174) and GW (*P* = 0.004, OR = 0.755, 95%CI: 0.625–0.912) were independent factors for PH-related complications. LRU was a risk factor, whereas GW was a protective factor (Table 2).

**Table 2:** Multivariate logistic regression analysis

Variable	Group	B	S. E	Wald	*P*-value	OR	95%CI
Age (years)		0.110	0.067	2.668	0.102	1.116	0.978–1.274
Length of the reconstructed urethra (cm)		1.223	0.215	32.392	<0.001	3.396	2.229–5.174
Glans width (mm)		-0.281	0.096	8.501	0.004	0.755	0.625–0.912
Season of surgery		-0.046	0.174	0.069	0.793	0.955	0.680–1.343
Degree of penile curvature	Moderate (30–45°)^*^						
	Severe (> 45°)	0.175	0.408	0.183	0.669	1.191	0.535–2.649
PH with prostatic utricle	No^*^						
	Yes	0.356	0.362	0.968	0.325	1.428	0.702–2.902

*Abbreviations*: *95% CI* 95% confidence interval, *OR* odds ratio

^*^Reference

### Receiver operating characteristics analysis

**Fig. 4:**
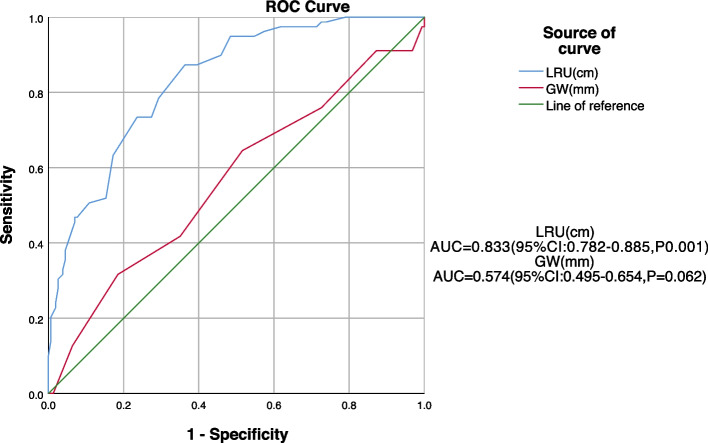
Receiver operating characteristic curve showing the predictive efficacy of independent factors for postoperative complications. Abbreviation: GW, glans width; LRU, length of the reconstructed urethra; ROC, receiver operating characteristic

In predicting complications after hypospadias repair by LRU and GW, no statistically significant threshold was observed for GW (*P* = 0.062). The optimal LRU threshold was 4.45 cm with an area under the curve of 0.833, sensitivity of 0.873, and specificity of 0.873 (*P*<0.001, OR = 3.396, 95%CI: 2.229–5.174; Table 3 and Fig. 4).

**Table 3:** Efficacy of independent factors in predicting postoperative complications

Indicators	Threshold	Sensitivity	Specificity	Standard error	Youden index	AUC	P	95% CI
Glans width	12.5	0.828	0.316	0.041	0.144	0.574	0.062	0.495–0.654
Length of the urethra	4.45	0.873	0.637	0.026	0.510	0.833	< 0.001	0.782–0.885

*Abbreviations*: *95% CI* 95% confidence interval, *AUC* area under the receiver operating characteristic curve

## Discussion

In pediatric urology, PH repair is the most challenging procedure [21]. It involves correction of abnormal penis curvatures, reconstruction of the urethra with its orifice positioned at the glans, and ensuring that the external genitals have a healthy appearance. In this study, we analyzed patients who underwent one-stage repair through disconnection of the urethral plate. The first operation succeeded in achieving satisfactory appearance in 157 cases (66.5%).

In our study, patients in the no-complication group were younger than those in the complication group. In the study by Dale et al. [22], in 98 hypospadias surgeries, no complications occurred in patients younger than 2 years, whereas 7 complications (11%) occurred in children older than 2 years. As penile erection, pain, and urethral discharge increase with age, the European Association of Urology recommends surgery at a younger age (6–18 months). According to Bai et al. [23], 76.6% of Chinese children undergo surgery after 18 months of age. In the current study, the median age at surgery was above 18 months, and topical androgen use increased the age of surgery in some instances. In addition to reducing surgical complications, early surgical treatment also has less impact on children’s mental health, preventing low self-esteem, introversion, and social withdrawal [24]. It may be difficult to determine the nature of developmental complications due to delayed surgical remodeling, even if successful [24]. The term hypospadias is still unknown to many families, especially in rural or remote areas, and increasing awareness regarding the condition remains crucial [23].

An important feature of PH is penile curvature, which is difficult to treat [11]. Nearly three-quarters of our patients had curvatures of > 45°, with severe penile curvatures requiring longer LRUs after urethral plate disconnection. This affects the blood flow to the flaps, and necessitates the creation of more flaps to form the new urethra. For surgery of moderate-to-severe curvatures, Shahin et al. [25] proposed measuring the urethra and flap accurately to mark the surgical site and avoiding cutting the flap too long or too short to prevent unnecessary trauma.

PH with prostatic utricle is highly prevalent in PH [26]. Devine et al. [27] found that 14% of hypospadias involved the prostatic utricle. Only a few studies have examined whether the prostatic utricle affects the complication frequency following hypospadias repair. Preoperatively, PH with prostatic utricle may be asymptomatic but likely increases the urethral curvature, flow resistance, and residual leakage of the prostatic utricle postoperatively, causing urinary tract infections, urinary retention, epididymitis, and difficulties urinating [28], which adversely affect surgical wound healing.

In patients with a short penis, small glans, and small urethral plate, androgens may be used preoperatively to increase the GW and support penile development [29, 30]. GW is an independent factor of complications following hypospadias repair [31]. One study reported a 14.9% reduction in complications after androgen therapy for every 1-mm increase in GW [32]. Bush et al. [33] found that a GW of < 14 mm is an independent risk factor for hypospadias reoperation. Our study confirms that GW is an independent influencing factor for complications after PH repair.

Likewise, LRU is also an independent risk factor of complications of PH. According to Zhou et al. [6], disconnection of a urethral plate < 4.55 cm is significantly associated with PH complications, which is similar to the 4.45-cm LRU threshold determined in our study. Reconstruction of long urethras require greater blood supply and higher levels of surgical skills. Moreover, insufficient tissue covering material can result in higher complication rates.

Complication rates were not significantly different among the Duckett, Duckett + Duplay, and modified Koyanagi procedures. The Duckett/Duplay procedures require greater surgical skill and flap quality, whereas the modified Koyanagi procedure can be used when the flap is not sufficiently long, uneven, or irregular. According to Acimi et al. [34], satisfactory surgical success rates can be achieved by selecting the appropriate approach based on the intraoperative situation.

In our study, PH repair had a complication rate of 33.5%, which was lower than that of 43.7% in the study by Fang et al. study [11], but significantly higher than that of 23.4% associated with distal hypospadias repair According to our results, surgical complications can be prevented by performing surgery at an earlier age, increasing GW using topical androgens, and paying particular attention to PH cases with the prostatic utricle, severe penile curvatures, and overly long urethras. Choosing the appropriate surgical approach, cutting the appropriate flap depending on the degree of development of the penis, urethral plate, and foreskin are crucial considerations during the procedure, in addition to the surgeon’s skills and experience. Postoperative complications may be reduced by introducing newer surgical materials; however, for the foreseeable future, PH will remain challenging for pediatric urologists [35, 36].

One of the limitations in the study is the retrospective nature of it.Our study was limited by the geographical and ethnic biases of single-center studies and the bias of choosing either Duckett, Duckett + Duplay, or modified Koyanagi procedures. Lastly, the follow-up period was too short and lacked data on reoperation, thereby hampering the ability to judge the study results.

## Conclusions

Surgical PH treatment remains difficult and carries a high complication risk. However, an experienced surgeon with sufficient surgical experience can achieve good results through one-stage hypospadias repair with urethral plate disconnection. LRU and GW are independent influencing factors for hypospadias complications.

## Data Availability

The datasets used and/or analysed during the current study available from the corresponding author on reasonable request.

## Abbreviations

BMI: Body mass index
CI: 95% Confidence interval
GW: Glans width
LRU: Length of the reconstructed urethra
OR: Odds ratio
PH: Proximal hypospadias
